# Cytological Grading of Lymphocytic Thyroiditis and Its Correlation With Biochemical Parameters: An Experience From a Tertiary Care Center in North India

**DOI:** 10.7759/cureus.58669

**Published:** 2024-04-21

**Authors:** Danish Ashraf, Poonam Sharma, Rajat Gupta, Subhash Bhardwaj

**Affiliations:** 1 Pathology, Sher-e-Kashmir Institute of Medical Sciences, Srinagar, IND; 2 Pathology, All India Institute of Medical Sciences, Vijaypur, Jammu, IND; 3 Pathology, Government Medical College, Jammu, Jammu, IND

**Keywords:** hypothyroidism, thyroid profile, cytological grading, thyroiditis, fine needle aspiration cytology

## Abstract

Background: Chronic lymphocytic thyroiditis is the most common cause of acquired hypothyroidism. The clinical management of thyroid nodules, with or without chronic lymphocytic thyroiditis, mainly depends on clinical data, ultrasonography, and fine-needle aspiration cytology (FNAC), the latter being the gold standard for the pre-surgical diagnosis of thyroidal nodules. The grading of chronic lymphocytic thyroiditis can be divided into three categories. The spectrum of the thyroid profile can be correlated to the cytological diagnosis of chronic lymphocytic thyroiditis.

Aim: This study aims to study the cytomorphology of various grades of chronic lymphocytic thyroiditis and its correlation with the hormonal profile.

Methods: In this study, 44 patients with a diagnosis of lymphocytic thyroiditis on FNAC were included. The cases of lymphocytic thyroiditis were graded cytomorphologically, and correlation with the thyroid hormone profile was done.

Results: The majority of the patients were between 16 and 30 years age group, with a female predominance. The majority of the patients presented with diffuse enlargement of the thyroid gland. The maximum number of cases was graded in the grade II cytological category (70.46%). A hypothyroid profile was present in 63% of patients, followed by an euthyroid profile. The majority of patients with grade II thyroiditis also had a hypothyroid profile. However, no significant association was found between cytological grading and hormonal status.

Conclusions: Cytological grading is a clear, easy-to-use diagnostic tool for confirmation of lymphocytic thyroiditis. However, the cytological grades show no statistically significant correlation with thyroid hormonal status. Lymphocytic thyroiditis should be diagnosed with a multidisciplinary approach, as clinical features and hormonal profile when used alone may result in a missed diagnosis.

## Introduction

Thyroid disorders rank among the most prevalent endocrine conditions globally. According to diverse studies on this subject, it is estimated that approximately 42 million individuals in India are affected by thyroid diseases [1]. Chronic lymphocytic thyroiditis is the cause of goitre and acquired hypothyroidism in a large population. Hakaru Hashimoto initially characterized Hashimoto's thyroiditis (HT) in 1912 [2], using the term as a synonym for chronic lymphocytic thyroiditis, and saw that the thyroid showed intense lymphocytic infiltrate, which he named struma lymphatosum, which was later named HT. In HT, the immune system erroneously targets the thyroid gland. This autoimmune disorder entails the stimulation of T and B cells directed against thyroid-specific proteins, thyroglobulin (TG), and thyroid peroxidase (TPO), resulting in inflammation and thyroid tissue damage. The stimulation of these immune cells may lead to the overproduction of reactive oxygen species (ROS) via the enzyme nicotinamide adenine dinucleotide phosphate (NADPH) oxidase, contributing to the pathology of HT [3].

Although clinical examination, hormonal profile, and ultrasonography (USG) of the thyroid gland are commonly used in the evaluation of both diffuse and solitary thyroid swellings, fine-needle aspiration cytology (FNAC) for the thyroid has become a widely accepted gold standard procedure in outpatient departments for assessing the pre-surgical diagnosis of thyroidal nodules with or without chronic lymphocytic thyroiditis. Besides FNAC, cytology using the non-aspiration technique is also increasingly becoming popular in the evaluation of thyroid nodules.

Chronic lymphocytic thyroiditis can be graded cytologically, and the grading system proposed by Bhatia et al. is commonly accepted for reporting. In this system, grade 1 is considered mild and is identified by a few lymphoid cells infiltrating the follicles/increased number of lymphocytes in the background with a few Hurthle cells (follicular-derived epithelial cells with oncocytic cytology). Grade 2, or moderate, is characterized by lymphocytic infiltration or mild lymphocytic infiltration with Hurthle cell clusters/giant cells/anisonucleosis. Grade 3, or florid, is described as lymphocytic inflammation with germinal centre formation and very few recognizable follicular cells, Hurthle cells, on smear [2].

In chronic lymphocytic thyroiditis, there is an elevation in thyroid-stimulating hormone (TSH) levels, a reduction in free T4 (FT4) levels, and decreased free T3 (FT3) levels. In the later stages, approximately 25% of cases may exhibit normal FT3 levels. The concentration of TPO antibodies is associated with the extent of lymphocytic infiltration in lymphocytic thyroiditis.

In a developing country like ours with limited resources, FNAC is the most basic and effective modality to diagnose with accuracy the common thyroid conditions, especially chronic lymphocytic thyroiditis. Cytological grading of HT on FNAC smears is a systematic approach to categorizing the disease based on the cytomorphological features observed [2,4,5]. However, the clinical relevance of cytological grading in predicting the biochemical severity of the disease remains unclear. Studies have shown no significant statistical correlation between cytological grades and levels of antithyroid antibodies such as antithyroid peroxidase antibody (ATPO) and antithyroglobulin antibody (ATG), as well as TSH [2,4,5]. This lack of correlation suggests that while cytological grading is useful for diagnosing HT, it may not be a reliable indicator of the biochemical severity of the disease. The rising trend of HT, either due to dietary iodine supplementation, improved diagnostic modalities, or both, has also been noted [2]. This increase in incidence raises questions about the potential role of iodine intake and other environmental factors in the pathogenesis of HT and whether these factors influence the correlation between cytological grading and biochemical parameters. Given these gaps in research, the present study aims to address whether the cytological grading of lymphocytic thyroiditis correlates with biochemical parameters. This question is pivotal in understanding the clinical implications of cytological grading and whether it can be used as a reliable predictor of disease severity and management in patients with HT.

## Materials and methods

Study design

This descriptive-analytical prospective study was conducted in the Department of Pathology at a tertiary care institute in North India for a duration of one year. The primary objective was to investigate the cytological grading of lymphocytic thyroiditis and its correlation with various biochemical parameters, including hormonal profiles.

Participants selection

The study recruited 44 consecutive patients attending the FNAC clinic from November 1, 2020, to October 31, 2021, who were clinically presumed to have chronic lymphocytic/autoimmune thyroiditis. The inclusion criteria mandated written consent from each patient for inclusion in the study. Exclusion criteria included patients on medication that could interfere with thyroid function and cases where smears were diluted with excessive blood.

Data collection

Clinical and Biochemical Assessment

Patients underwent clinical and biochemical evaluations. This included the estimation of thyroid hormones (T3, T4, and thyroid-stimulating hormone [TSH]), with normal ranges for T3, T4, and TSH set at 0.52-1.85 ng/ml for T3, 4.4-10.8 μg% for T4, and 0.4-6.1 μIU/ml for TSH, respectively [6].

Fine-Needle Aspiration Cytology

FNAC of the thyroid was performed from several locations without USG guidance. The procedure involved a maximum of two to four attempts per patient to procure adequate material. Smears were prepared and stained with May Grunwald Giemsa (MGG) and Papanicolaou (PAP) stains. In cases where smears were deemed unsatisfactory, repeat aspiration was performed. A minimum of two good smears displaying features of lymphocytic thyroiditis were selected for the study. Two independent cytologists evaluated the smears.

Cytological Grading

The cytological diagnosis was based on qualitative criteria, including the presence of lymphocytes and plasma cells infiltrating the thyroid follicles, an increased number of lymphocytes in the background, and other features such as Hurthle cell change, multinucleated giant cells, epithelioid cell clusters, anisonucleosis, or interlobular fibrosis. Quantitative assessment of thyroiditis was performed using a cytological grading system that considered the number of lymphocytes infiltrating the gland, the degree of destruction caused, and associated features like Hurthle cell change, giant cells, anisonucleosis, etc. Cytological grading was conducted according to the criteria proposed by Bhatia et al. [2].

## Results

Cytological characteristics are a basic tool in the diagnosis of chronic lymphocytic thyroiditis. In this study, the majority of the patients were between 16 and 30 years old, accounting for 40% of the cases. The majority of the cases were female, accounting for 93% of the cases with an F:M ratio of 13:1. 77% of the patients presented with diffuse enlargement of the thyroid gland. Various cytological features of chronic lymphocytic thyroiditis are tabulated in Table 1. Most of the cytological smears were cellular, with lymphocytic infiltration. Anisokaryosis with Hurthle cell change formed the predominant diagnostic cytological component.

**Table 1:** Various cytological features of chronic lymphocytic thyroiditis.

S. No.	Cytological features	Total cases	Percentage
1	Thyroid epithelial cells	44	100%
2	Follicular lymphocytic infiltration	44	100%
3	Background lymphocytes	44	100%
4	Anisokaryosis	35	79.50%
5	Hurthle cells	34	77.27%
6	Giant cells	15	34.09%
7	Epitheliod like cells	7	15.90%
8	Granuloma	3	6.81%
9	Germinal center formation	5	11.36%
10	Colloid	13	29.54%
11	Histiocytes engulfing colloid	7	15.90%
12	Plasma cells	5	11.30%

The maximum number of cases (31 cases) were graded as a grade II cytological category (70.46%) based on the classification proposed by Bhatia et al. [2] (Table 2). Grade I was the second most common category in our study, accounting for eight cases (18.18%).

**Table 2:** Cytological grading of cases (n=44).

S.no	Cytological grade	No. of cases	Percentage
1	Grade I	8	18.18%
2	Grade II	31	70.46%
3	Grade III	5	11.36%
4	Total	44	100%

A thyroid profile was available in all cases. On hormonal evaluation, 28 patients (63%) had a hypothyroid profile (Table 3).

**Table 3:** Thyroid hormone status in all the cases (n=44).

Thyroid status	No. of cases	Percentage %
Euthyroid	9	20.45%
Hyperthyroid	7	15.90%
Hypothyroid	28	63.64%
Total	44	100%

Table 4 shows that the majority of cases with a hypothyroid profile fell into the grade II category of thyroiditis (19 cases; 61.2%). The cytological grading of chronic lymphocytic thyroiditis showed no significant association with hormonal status (p-value > 0.05).

**Table 4:** Cytological grades of lymphocytic thyroiditis and hormonal profile in each category (n=44).

Cytological grading	Grade I no. (%)	Grade II no. (%)	Grade III no. (%)	Total no. (%)
Hypothyroid	6 (75)	19 (61.2)	4 (80)	29 (65.9)
Hyperthyroid	0 (0)	7 (22.5)	0 (0)	7 (15.9)
Euthyroid	2 (25)	5 (16.12)	1 (20)	8 (18.18)
Total	8 (100)	31 (100)	5 (100)	44 (100)

## Discussion

Hashimoto's thyroiditis, also known as chronic lymphocytic thyroiditis, is an autoimmune disorder that is frequently diagnosed through fine-needle aspiration cytology. It is characterized by the infiltration of the thyroid gland by lymphocytes, leading to glandular destruction and, often, hypothyroidism. The cytological grading of HT is an important diagnostic criterion that is based on the extent of lymphocytic infiltration and the presence of other cellular changes. The current study aimed to cytologically grade lymphocytic thyroiditis and correlate these cytological grades with the thyroid profile.

Forty-four patients formed the material for our study. The majority of the patients were seen in the third decade. Our findings are similar to those published in the literature [2,4,5]. In our study, female predominance was seen with a female/male ratio of 13:1, similar to other studies [2,4,5,7]. In young patients, the prevalence of the disease can be attributed to iodine deficiency in non-coastal regions, a situation that persists despite the implementation of the national iodine deficiency disease control programme. Conversely, in the elderly, the disease may manifest even in areas with sufficient iodine levels. Numerous authors have associated the heightened incidence of Hashimoto's thyroiditis, particularly in coastal areas, with an excess intake of iodine or in populations with sufficient iodine intake [2,8-10].

The most common presenting symptom was the diffuse enlargement of the thyroid gland, which was seen in over 70% of the cases. These observations were comparable to those made by Singh et al. [10], Iha et al. [11], and Chandanwale et al. [7]. However, this figure is notably elevated when contrasted with the study conducted by Bhatia et al. [2] and Friedman et al. [12]. The authors suggest that nodular disease typically signifies the initial phases of Hashimoto's (lymphocytic) thyroiditis. Patients, however, often seek medical attention in the advanced stages of the disease, where clinical and hormonal alterations have already manifested and the patient presents clinically with diffuse thyroid swelling.

The clinical presentation of HT can vary from diffuse enlargement of the thyroid gland to nodular disease. While the cytological grading provides a snapshot of the autoimmune activity within the thyroid gland, it does not necessarily reflect the functional status of the gland. For instance, patients with early-stage HT may present with nodular disease and normal TSH levels despite having elevated antithyroid antibodies [4,5].

In 35 (79%) cases, T3 values were within the normal range. Normal T3 levels showed no specific correlation with the thyroid status of the patient. In comparison to T3 levels in this study, normal T4 levels were seen in only 11 (25%) cases. TSH, which is the most specific and common marker for the assessment of the thyroid profile of the patient, was raised in 28 (63.6%) of the cases. Patients in our study were categorized into three groups based on thyroid function tests that included hypothyroid, euthyroid, and hyperthyroid. The thyroid function test showed that the majority of the cases belonged to the hypothyroid group, representing 63.6% of the total. The second most common category in our study was euthyroid status, seen in 9 (20.45%) cases. Hyperthyroid status was observed in 7 (15.9%) cases. These values correlated with the studies done by Kumar et al. [13] and Singh et al. [10]. However, the study by Jayaram et al. reported euthyroid status in 55% of cases in their study [14].

**Figure 1:**
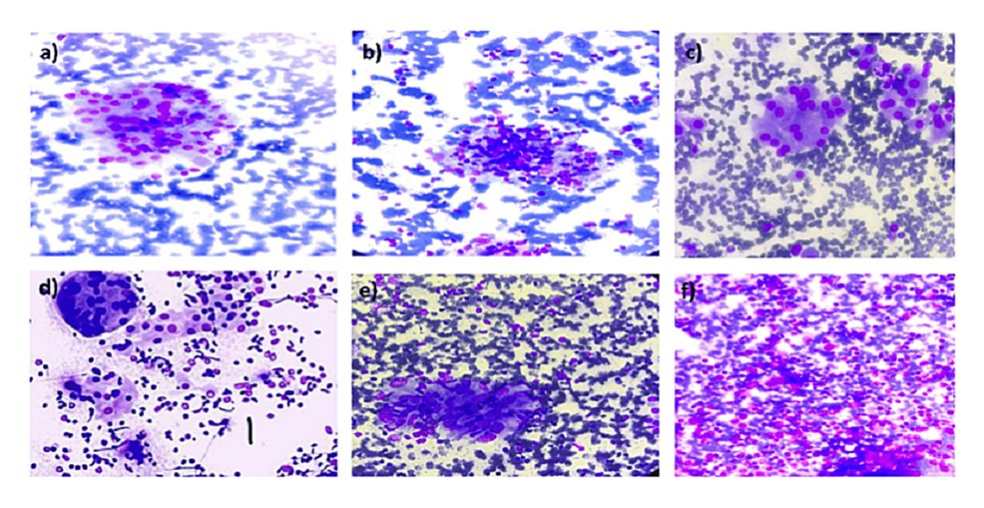
Grading system for chronic lymphocytic thyroiditis. (a) Grade I lymphocytic thyroiditis showing follicular epithelial cells infiltrated by lymphocytes (400×, MGG); (b) grade II chronic lymphocytic thyroiditis showing follicular cells infiltrated with lymphocytes along with Hurthle cell change (400×, MGG); (c) grade II lymphocytic thyroiditis showing Hurthle cell change (400×, MGG); (d) grade II lymphocytic thyroiditis showing Hurthle cell change with multinucleated giant cell with lymphocytic infiltration (400×, MGG); (e) grade II lymphocytic thyroiditis showing aggregates of cells with epitheloid morphology-simulating Granuloma formation (400×, MGG); (f) florid lymphocytic infiltrate in case of grade III lymphocytic thyroiditis (400×, MGG).

The application of the grading system for chronic lymphocytic thyroiditis, as per the criteria established by Bhatia et al. [2], demonstrated notable consistency, with a high concordance rate observed across all the studies. These grades were subsequently subjected to statistical correlation with the hormonal profile. Applying these simple, practical, and easily applicable criteria, 44 cases of lymphocytic thyroiditis in the present study were categorized into grade I, grade II, and grade III thyroiditis, consisting of 18.1%, 70.45%, and 11.36% cases, respectively (Figure 1). Based on cytomorphological features, a moderate to high yield was seen in 90% of cases. Follicular epithelial cells, lymphocytic infiltration, and background lymphocytes were seen in 100% of the cases. Similar results were also reported by Kumar et al. [13] and Iha et al. [11]. Anisokaryosis and Hurthle cell changes were seen in 79.5% and 77.27% of cases, respectively, correlating with the study done by Iha et al. [11]. Colloid was found in 29.5% of cases, which correlated with the study done by Kumar et al. [13]. In our study, grade I thyroiditis was seen in 8 (18.1%) cases only, and these features correlate with the study of Singh et al. [10]. We found hypothyroidism in 75% of cases of grade I thyroiditis and euthyroidism in the remaining cases [10]. No significant association was seen between grade I thyroiditis and hormonal profile (p-value > 0.05), and these findings are comparable with other studies [5,10,11].

Category II formed the majority of the cases in our study, forming 70.45% of the total, similar to previous studies [5,10,11,15]. In grade II lymphocytic thyroiditis, 61% of cases were hypothyroid, 22.58% were hyperthyroid, and 16.42% were euthyroid. Our results were in agreement with previous studies [15,16]. On statistical analysis, no significant association was seen between the grade II chronic lymphocytic thyroiditis and hormonal profile, similar to prior studies [5,10,11,15].

In our study, grade III lymphocytic thyroiditis was found in 5 (11.3%) cases. Cases were categorized on the basis of cytological features of florid lymphocytic inflammation with germinal centre formation and very few follicular cells left. Our numbers were in correlation with the studies done by Bhatia et al. [2] and Iha et al. [11]. Grade III chronic lymphocytic thyroiditis had hypothyroid status in 80% of cases, with the rest being euthyroid based on the thyroid profile. These findings correlated with the other studies done by Gupta et al. [15] and Uma et al. [16]. The results of our study did not show any association between grade III lymphocytic thyroiditis and hormonal profile, which is in agreement with the previous studies [5,11,15].

In the past, the grading of thyroiditis has traditionally been conducted on histological specimens, focusing on the number of lymphocytic foci per standard representative section. In contrast, only a limited number of researchers have undertaken grading on cytology smears. Following various statistical analyses, no significant correlation was found between cytomorphological grades and the thyroid profile, with a p-value greater than 0.05 (0.213 in our study). The cytological grading of HT, as proposed by Bhatia et al. [2], is a systematic approach to categorize the disease based on the cytomorphological features observed in FNAC smears. This grading system has been used to correlate the extent of lymphocytic infiltration with clinical, biochemical, ultrasonographic, and radionuclide parameters. However, studies, including the current one, have consistently shown no significant statistical correlation between cytological grades and levels of antithyroid antibodies such as ATPO and ATG, as well as TSH [2,5,13,17]. This lack of significance may be attributed to factors affecting grading on FNAC smears, such as dilution by blood, FNAC technique, and the number of aspirations performed. Moreover, the collection of aspirates from a very small portion of the thyroid gland may not always fully represent the pathology.

The lack of correlation suggests that while cytological grading is useful for diagnosing HT, it may not be a reliable indicator of the biochemical severity of the disease. This is further supported by the observation that many patients with HT have elevated ATPO and ATG levels, irrespective of the cytological grade, with normal TSH values [4]. This indicates that the autoimmune process, as evidenced by the presence of antithyroid antibodies, may precede the clinical and biochemical manifestations of thyroid dysfunction.

One of the limitations of the current study, as well as previous ones, is the sample size. Larger studies may be required to definitively establish the relationship between cytological grades and biochemical parameters. Additionally, longitudinal studies could provide insights into the progression of HT and the development of hypothyroidism over time.

Future research should also focus on the potential role of other biomarkers in predicting the course of HT and the response to therapy. Understanding the molecular mechanisms underlying the autoimmune process in HT may lead to the identification of novel therapeutic targets and more personalized treatment strategies.

## Conclusions

The study was a comprehensive cytological assessment of the cytomorphological features of chronic lymphocytic thyroiditis, with a variety of particular cytological features within various grades seen in the 44 cases studied in our cross-sectional study, and then we correlated every single case with its hormonal profile. Cytological grading is a clear, easy-to-use diagnostic tool for the confirmation of lymphocytic thyroiditis. However, the cytological grades show no statistically significant correlation with thyroid hormonal status. Lymphocytic thyroiditis should be diagnosed with a multidisciplinary approach, as clinical features and hormonal profile, when used alone, may result in a missed diagnosis.
